# Cystatin C prevents tissue injury after lung transplantation

**DOI:** 10.26508/lsa.202503312

**Published:** 2025-12-16

**Authors:** Henu Kumar Verma, Carmela Morrone, Salome RT Rehm, Aicha Jeridi, Natalia F Smirnova, Ali Doryab, Stefanie AI Weiß, Alexey Dashkevich, Otmar Schmid, Carlo Mümmler, Michael Gerckens, Thomas M Conlon, Matthias Hecker, Nikolaus Kneidinger, Dieter E Jenne, Ali Önder Yildirim

**Affiliations:** 1 Institute of Lung Health and Immunity (LHI), Comprehensive Pneumology Center (CPC), Helmholtz Munich, Member of the German Center for Lung Research (DZL), Munich, Germany; 2 Inserm, Institut des Maladies Métaboliques et Cardiovasculaires, Universite de Toulouse, Toulouse, France; 3 Department of Cardiac Surgery, Leipzig Heart Center, Leipzig, Germany; 4 Department of Internal Medicine II, Universities of Giessen and Marburg Lung Center (UGMLC), Excellence Cluster Cardiopulmonary Institute (CPI), Member of the German Center for Lung Research (DZL), Justus-Liebig University, Giessen, Germany; 5 Department of Internal Medicine V, Ludwig-Maximilians University (LMU) Munich , CPC-M, Member of the German Center for Lung Research (DZL), Munich, Germany; 6 Division of Respiratory Medicine, Department of Internal Medicine, Lung Research Cluster, Medical University of Graz , Graz, Austria; 7 Max Planck Institute of Biological Intelligence , Planegg, Germany; 8 Institute of Experimental Pneumology, Ludwig-Maximilians University (LMU) , Munich, Germany

## Abstract

Lung transplantation (LTx) offers life-saving therapy for end-stage lung disease, yet primary graft dysfunction (PGD) and bronchiolitis obliterans syndrome remain major complications, which limit long-term outcomes. The risk of PGD increases with prolonged storage and reperfusion injury. This study investigated the protective effects of cystatin C (CysC), a cysteine protease inhibitor, against PGD, through neutralization of cathepsin B activity. LTx recipients exhibited reduced levels of CysC and greater lung damage reflected by increased γ-H2AX and ACSL4 expression, correlating with poorer outcome. Using an orthotopic LTx model, we engineered CysC-Alb, an albumin-fused, cell-permeable CysC derivative for enhanced lung preservation. Adding CysC-Alb to preservation solutions during cold storage and ex vivo lung perfusion improved oxygenation, reduced DNA damage, and minimized cell death, particularly in alveolar type 2 cells. In murine grafts, CysC-Alb decreased γ-H2AX and ACSL4 expression, markers of DNA damage and ferroptosis, respectively. These findings highlight CysC-Alb as an effective additive to mitigate early pulmonary dysfunction after LTx and improve lung graft viability and transplantation outcomes.

## Abstract (precis)

Cystatin C added to the preservation solution during cold storage and ex vivo lung perfusion of donor lungs results in improved tissue preservation and protection against lung tissue injury.

## Introduction

Lung transplantation (LTx) remains the definitive treatment option for patients with end-stage lung disease. However, long-term outcomes are significantly impacted by early complications like primary graft dysfunction (PGD), which typically develops within 72 h after surgery. PGD is marked by tissue edema, neutrophil infiltration, and impaired pulmonary function, making it a critical risk factor for morbidity and early mortality after transplantation (Christie et al, 2005; Kreisel et al, 2011; Yusen et al, 2013). To prepare for implantation, the donor lung is kept at 4°C to slow metabolic processes (Lepoittevin et al, 2022). Upon reperfusion with warm blood in the recipient, inflammatory responses and the release of inflammatory mediators can lead to irreversible lung tissue damage (Zimmerman & Granger, 1994). It has been suggested that lysosomal cysteine proteases, which are synthesized as inactive proenzymes in the endoplasmic reticulum and later activated within lysosomes, are released during both cold storage and warm perfusion (Lalmanach et al, 2015). Depending on their location, these proteases facilitate various physiological functions, such as lysosomal protein degradation, antigen processing, and selective protein modification.

Recently, we have identified the cysteine protease, cathepsin B (CatB), as a key contributor to inflammation and pathological changes post-LTx (Morrone et al, 2021). CatB played a crucial role in initiating the cascade of events that lead to early lung graft injury, highlighting its potential as a therapeutic target. Cystatin C (CysC) is a key endogenous inhibitor of cysteine proteases, including CatB (Wallin et al, 2013). CysC plays a protective role, inhibiting extracellular proteases released from damaged tissue or tumor cells (Rudzinska et al, 2019). However, the therapeutic potential of small proteins like CysC may be limited by their rapid clearance from the circulation and potentially reduced accumulation at extravascular sites. Indeed, the half-life of endogenous CysC in the bloodstream is 2 h (Filler et al, 2005). One strategy to extend the circulatory half-life and tissue distribution of such proteins is albumin fusion, which binds to the neonatal Fc receptor (FcRn) and enables intracellular recycling (Schulte 2008; Strohl 2015). These albumin-fused proteins are protected from lysosomal degradation and can achieve an extended half-life beyond that of the native protein (Strohl 2015); indeed, the circulatory half-life of albumin is up to 21 d in humans (Chaudhury et al, 2006).

Because of the ongoing risk of PGD posttransplantation and the critical shortage of donor lungs, optimizing donor lung use remains a clinical priority. Although strict donor criteria historically limited the use of lungs from donors after brain death (DBD) and factors like trauma, pneumonia, or barotrauma, many centers now successfully transplant marginal donor lungs, and the use of DBD lungs has become standard practice. The estimate that ∼40% of previously discarded lungs could be used is already reflected in the increasing number of LTxs worldwide (Yu & Son, 2022). Ex vivo lung perfusion (EVLP) has transitioned from an experimental tool to a clinical application, enabling assessment and reconditioning of donor lungs (Wierup et al, 2006). However, its limitations have become apparent (Faccioli et al, 2024), and newer approaches such as static hypothermic storage at 10°C (Ali et al, 2021, 2023) and devices like the LUNGguard (Pontula et al, 2022; Haney et al, 2024) are emerging as alternatives in many transplant programs. Moreover, normothermic perfusion allows for the administration of therapeutic agents to the donor lung before implantation.

In this study, we aimed to explore whether cysteine protease–mediated tissue damage during lung storage could be reduced by introducing natural inhibitors into the preservation solution during extended cold ischemia and EVLP. First, we investigated the impact of reduced cathepsin B inhibitors in transplanted lungs, which may contribute significantly to the development of PGD. We further evaluated the therapeutic potential of targeting cysteine protease activity during EVLP as a strategy to protect against PGD. To achieve this, we genetically fused CysC with albumin (Alb), producing a fusion protein (CysC-Alb) that resists lysosomal degradation. Using an orthotopic left LTx model, we demonstrated that ischemic storage time could be extended to 18 h with improved posttransplant graft function when CysC-Alb was included in the preservation solution. In addition, CysC-Alb alleviates pulmonary dysfunction and reduces lung tissue edema, ultimately preserving lung function and improving graft viability post-LTx. This study confirmed the therapeutic potential of CysC-Alb in limiting early graft dysfunction, with promising implications for enhancing LTx outcomes.

## Results and Discussion

### Cystatin C levels are reduced early after LTx

Our study was initially designed to identify and categorize LTx recipients based on their clinical lung function decline over time. Bronchoalveolar lavage fluid (BALF) samples were obtained from the patients (n = 39) during bronchoscopy examination. Those without bronchiolitis obliterans syndrome (BOS) at the time of BALF sampling were classified as stable LTx (n = 24). Furthermore, patients with the chronic lung allograft dysfunction phenotype BOS were classified based on the decline in forced expiratory volume in 1 s (FEV1) and used to categorize lungs into BOS stages at the time of BALF sampling: pre-BOS (FEV1 > 90%, n = 28), BOS1 (FEV1 66–80%, n = 35), BOS2 (FEV1 51–65%, n = 33), and BOS3 (FEV1 < 50%, n = 31) (outlined in Table S1).

Table S1. Patient characteristics of lung transplant recipients.

**Figure 1.**
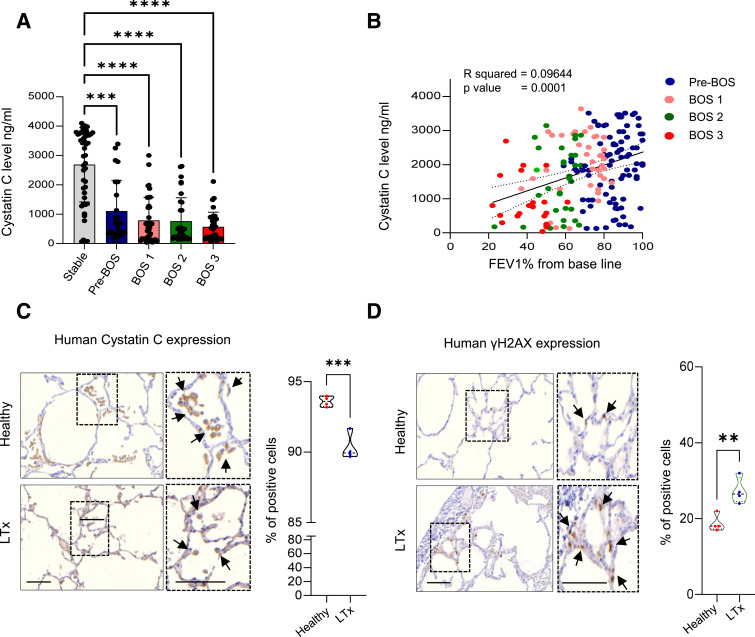
Cystatin C is reduced in the BALF and lung tissue post–lung transplantation (LTx). **(A, B)** CysC concentration determined by ELISA in human BALF from LTx recipients obtained serially posttransplantation. **(A)** Patients are stratified according to BOS severity at the time of sampling. **(B)** Linear regression mixed-effect correlation between BALF CysC and FEV1% in BOS patients at the time of sampling. **(C, D)** CysC immunohistochemical staining (brown signal in macrophages indicated by arrows) and (D) γ-H2AX immunohistochemical staining (brown signal in epithelial cells indicated by arrows), with hematoxylin counterstained (scale bars: 50 μm) in lung tissue sections from healthy and implanted lungs 2 h post-LTx (n = 4). The number of positive cells per 50 random fields of view was calculated as a percentage. **(A, C, D)** Statistical significance was measured using a Kruskal–Wallis test with Dunn’s multiple comparisons test (A) and unpaired two-tailed *t* test (C, D); **P < 0.01, ***P < 0.001, and ****P < 0.0001. Source data are available for this figure.

**Figure S1.**
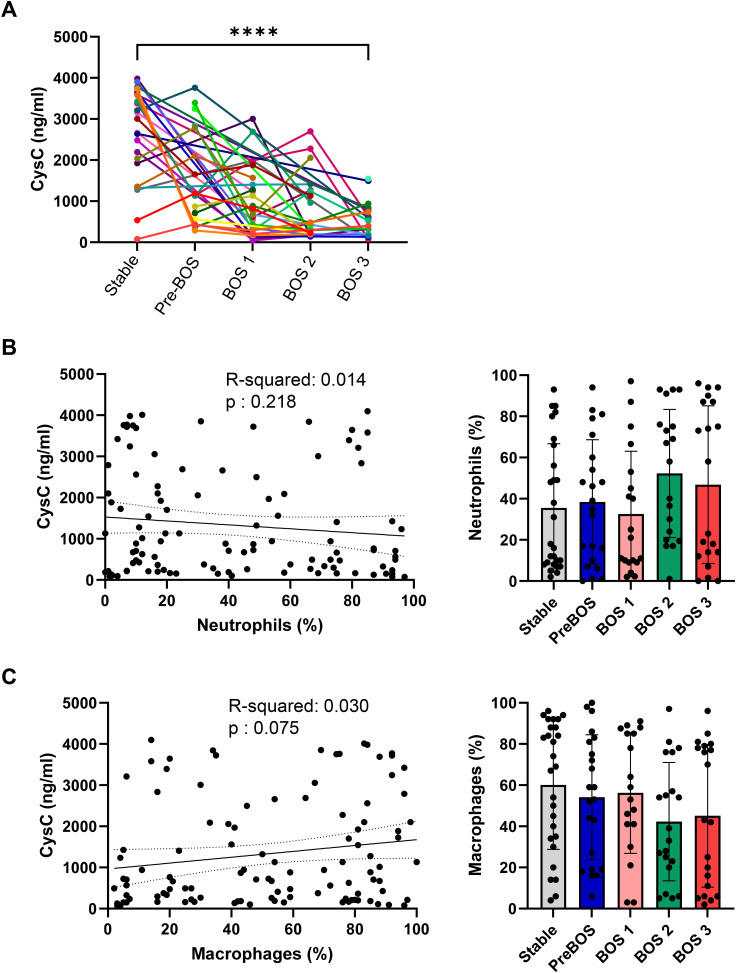
CysC concentration and the numbers of neutrophils and macrophages in the BALF of lung transplantation recipients. **(A)** CysC concentration determined by ELISA in human BALF from lung transplantation recipients obtained serially posttransplantation; individual patients shown (n = 39) are each represented by a different color and stratified according to BOS severity. ****P < 0.0001, two-way ANOVA with Dunnett’s multiple comparisons test. **(B, C)** Number of neutrophils (B) and macrophages (C) in human BAL from lung transplantation recipients (n = 36) obtained serially posttransplantation compared with CysC levels (left, linear regression analysis) and BOS stage (right, no significance, Kruskal–Wallis test). Source data are available for this figure.

**Figure S2.**
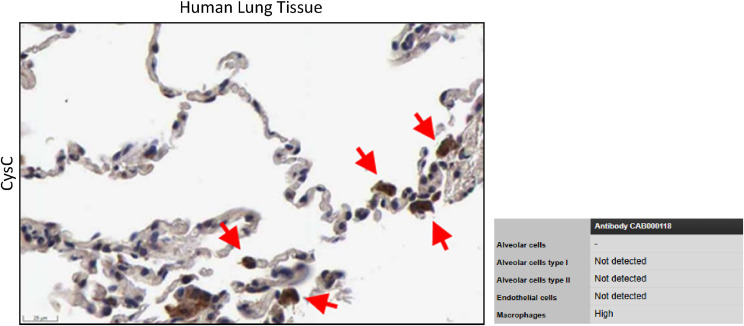
CysC expression in macrophages (red arrows) taken from the Human Protein Atlas v24.0 (scale bar: 25 μm), image: www.proteinatlas.org/ENSG00000101439-CST3/tissue/lung#img, image credit: Human Protein Atlas.

CysC is considered an important endogenous inhibitor of CatB (Morrone et al, 2021); we therefore assessed its levels in the BALF of lung transplant recipients using ELISA. Interestingly, our observations revealed a significant decline in CysC levels, which inversely correlated with FEV1% in the BALF of LTx recipients already at the early stage of BOS development (pre-BOS) and concurrent with increasing BOS stage (Figs 1A and B and S1A). There was, however, no correlation between the concentration of BALF CysC and the number of macrophages or neutrophils in the BALF at the time of sampling (Fig S1B and C). Likewise, there was no difference in the number of these cells in the BALF at differing BOS stages (Fig S1B and C). In line with a decline of CysC levels in the BALF, we observed a significant reduction in CysC levels in human lung tissue biopsies as early as 2 h post-LTx, compared with healthy lung controls upon immunohistochemical analysis (Fig 1C), with CysC predominantly localizing to the macrophages in human lung tissue (Figs 1C and S2) (Morrone et al, 2021). This decrease in CysC was accompanied by a notable increase of γ-H2AX–positive epithelial cells in the same lung tissue biopsies (Fig 1D), indicating an early cellular response to DNA double-strand breaks in the early transplanted lung tissue. These observations suggest that the early posttransplant period is critical, as rapid changes in CysC levels (*P* < 0.001) and an increased DNA damage response (*P* < 0.01) occur. The decreased expression of CysC and the concomitant increase in DNA damage in transplanted lungs underscore the importance of CysC after LTx and may potentially serve as a biomarker for early damage after LTx or as a target for therapeutic intervention.

### Cystatin C improves early LTx outcomes

**Figure 2.**
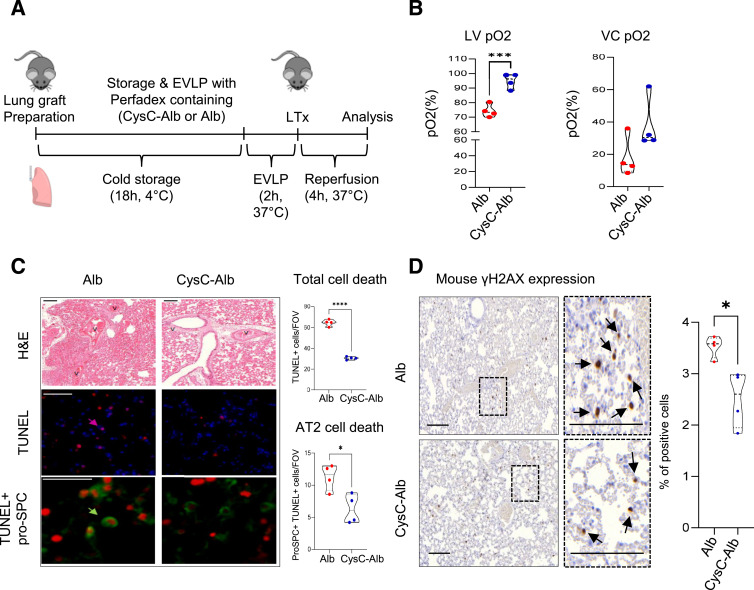
Early lung injury after lung transplantation (LTx) is ameliorated by cystatin C. **(A)** Experimental setup of donor lung storage, EVLP, and LTx in C57BL/6 mice. Preservation solutions were supplemented with 40 μg/ml of CysC-Alb or Alb. Mice were euthanized 4 h after LTx. **(B)** pO2 of the blood collected from the left ventricle and from the vena cava 4 h after LTx from the groups indicated (n = 4). **(C)** Representative pictures of H&E-stained lung tissue sections (scale bar: 200 μm) from transplanted lungs, and TUNEL (red) and proSPC (green) immunofluorescence staining (scale bar: 50 μm), individual lungs shown (n = 4). Quantification was given as the number of positive cells per random field of view. **(D)** γ-H2AX expression (brown signal, scale bars: 50 μm) in lung tissue sections from transplanted mouse lungs (n = 4). The number of positive cells per 50 random fields of view was calculated as a percentage. **(B, C, D)** Statistical significance was measured using an unpaired two-tailed *t* test (B, C, D); *P < 0.05, ***P < 0.001, and ****P < 0.0001. Source data are available for this figure.

**Figure S3.**
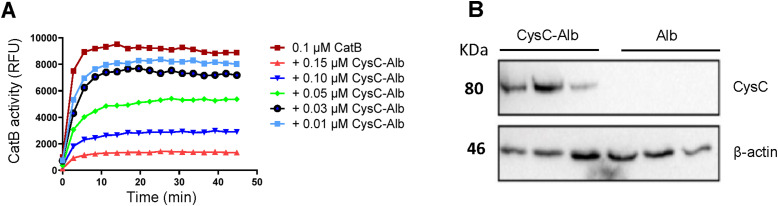
CysC-Alb profiling. **(A)** Relative activity of 0.1 μM recombinant CatB in the presence of increasing concentrations of CysC-Alb at the concentrations indicated. **(B)** Western blot analysis of CysC in total tissue of implanted lung 4 h after transplantation of donor lungs treated with preservation solution that was supplemented with 40 μg/ml of CysC-Alb or Alb (n = 3). Source data are available for this figure.

**Figure S4.**
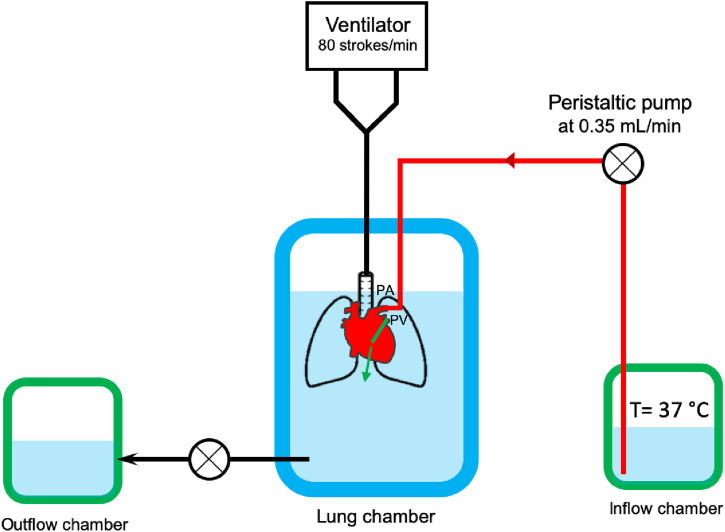
Ex vivo lung perfusion system. Lung is connected to a ventilation system (80 strokes/min) via the bronchus and the inflow and outflow cannulas via the pulmonary artery and vein; the lung is placed in a chamber containing Perfadex. Perfadex, which is kept at 37°C in the inflow chamber, reaches via a pump (0.35 ml/min) the lung through the pulmonary artery and is flushed out through the pulmonary vein in the lung chamber. Perfusate is collected in the outflow chamber.

**Figure S5.**
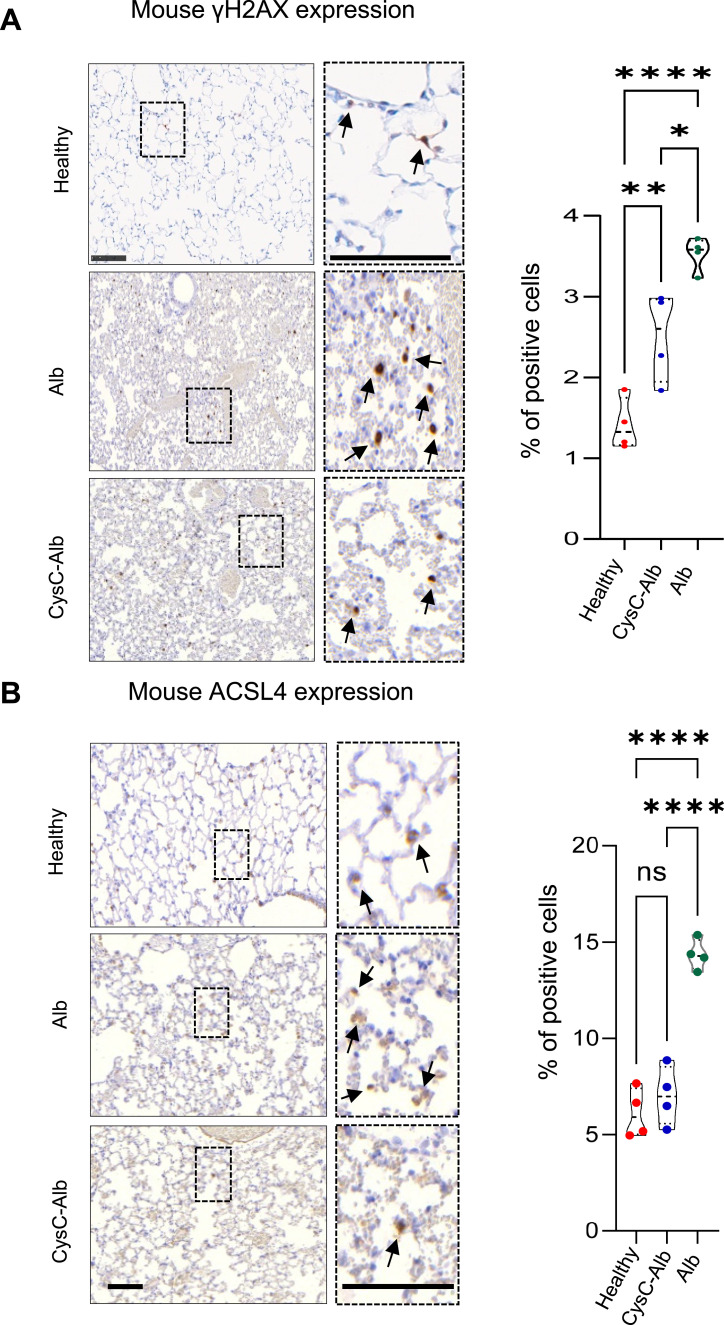
γ-H2AX expression and ACSL4 expression in lung tissue sections from transplanted and healthy control mice. **(A)** γ-H2AX expression and (B) ACSL4 expression (brown signal, scale bars: 50 μm) in lung tissue sections from transplanted and healthy control C57BL/6 mouse lungs (n = 4). The number of positive cells per 50 random fields of view was calculated as a percentage. Statistical significance was measured using an unpaired two-tailed *t* test; *P < 0.05, **P < 0.001, and ****P < 0.0001. Source data are available for this figure.

To investigate novel treatment options to prevent early LTx damage and dysfunction, we used a model of orthotopic LTx with syngeneic C57BL/6 grafts to mimic the induction of ischemia–reperfusion injury after extended graft storage in the cold without the complication of allorecognition (Fig 2A). Here, we developed a highly cell-permeable cysteine protease inhibitor named CysC-Alb, which possesses an increased circulatory half-life and strong inhibitory activity against CatB (Fig S3A). This inhibitor was specifically designed and synthesized to enhance the preservation conditions of donor lungs. By incorporating CysC-Alb into the preservation solution during cold storage and ex vivo lung perfusion (EVLP) (Fig S4), we aimed to enable extended storage durations and improved tissue preservation. Our findings revealed that ischemic storage times of lung transplants could be extended up to 18 h with improved transplant function posttransplantation when CysC-Alb was included in the preservation solution, which resulted in increased CysC in total lung tissue of the implanted lung (Fig S3B). The addition of CysC-Alb resulted in almost 30% higher blood oxygenation in the left ventricle (LV pO2, *P* < 0.001) and in peripheral regions (VC pO2, *P* = 0.097) compared with the control (Fig 2B). Furthermore, H&E staining of lung tissue showed significantly cleared airspace with less endothelial damage in the CysC-Alb treatment group compared with albumin alone (Fig 2C). Crucially, TUNEL staining revealed a substantial reduction of cell death in CysC-Alb–pretreated lungs with a specific protection in alveolar type 2 (AT2) cells as revealed by co-immunofluorescence staining with anti-proSPC (Fig 2C), which was accompanied by reduced DNA damage as indicated by a decrease in γ-H2AX expression (Fig 2D), but did not fully return to the levels observed in healthy C57BL/6 lungs (Fig S5A). Taken together, this suggests that the addition of CysC-Alb to the preservation solution prevents lung damage after LTx.

### Ferroptosis of AT2 cells is reduced by cystatin C

**Figure 3.**
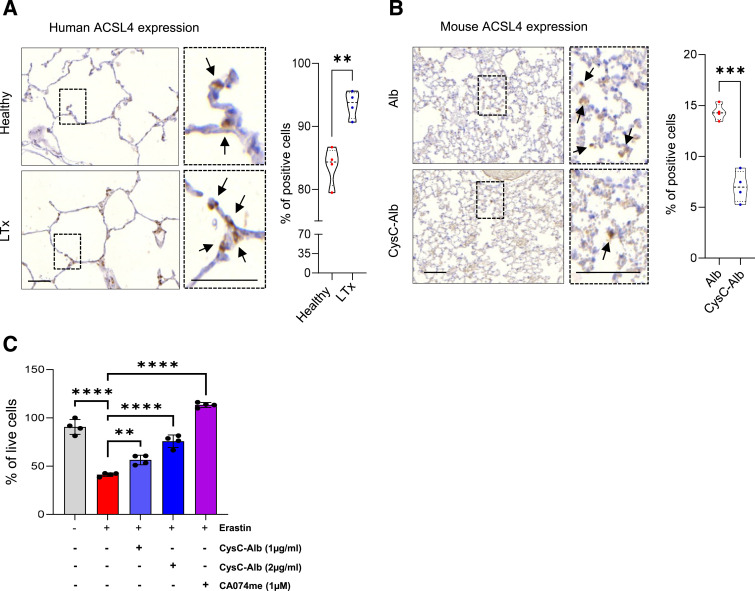
Cystatin C protects against epithelial cell death. **(A)** ACSL4 expression (brown staining, scale bars: 50 μm) in healthy and transplanted human lungs 2 h post–lung transplantation (n = 4). **(B)** ACSL4 expression (brown staining, scale bars: 50 μm) in mouse transplanted lungs 4 h post–lung transplantation, pretreated with preservation solutions that were supplemented with 40 μg/ml of CysC-Alb or Alb (n = 4). **(C)** Cell viability in MLE12 cells treated with erastin for 24 h in the presence or absence of CysC-Alb or CA074me. The bars represent the mean ± SD of four independent experiments. For all histological image quantifications, the number of positive cells per 50 random fields of view was calculated as a percentage. **(A, B, C)** Statistical significance was measured using an unpaired two-tailed *t* test (A, B) and one-way ANOVA with Bonferroni’s posttest (C); **P < 0.01, ***P < 0.001, and ****P < 0.0001. Source data are available for this figure.

Recently, we have highlighted the contribution of ferroptosis, a regulated form of necrotic cell death characterized by iron-dependent lipid peroxidation (Conrad & Pratt, 2019), to lung pathology, which is driven in part by up-regulation of the Acyl-CoA synthetase long-chain family member 4 (ACSL4) (Gunes Gunsel et al, 2022; Wang & Xia, 2023). Notably, ACSL4 expression increased in human lung tissue as early as 2 h post-LTx, compared with healthy lung controls (Fig 3A). Likewise, an increase was observed in murine transplanted lung, which was reduced upon the addition of CysC-Alb to the preservation solution (Fig 3B), returning to the levels observed in healthy C57BL/6 control lungs (Fig S5B), suggesting protection against ferroptotic cell death. In support, ferroptotic cell death induced by erastin treatment, which inhibits the cystine/glutamate antiporter system xc− leading to uncontrolled lipid peroxidation (Dixon et al, 2012), of in vitro–cultured murine MLE12 AT2-like cells, was reversed by cotreatment with CysC-Alb or the synthetic inhibitor of cathepsin B activity CA074me (Fig 3C), indicating cystatin C–cathepsin B axis as a potential regulator of lung ferroptosis after transplantation. Recent work also highlighted cathepsin B as a mediator of ferroptosis in mouse embryonic fibroblasts, proposing a mechanism of cathepsin B–driven functional changes in mitochondria and histone cleavage and chromatin degradation (Nagakannan et al, 2021). Indeed, we observed increased DNA damage as revealed by γ-H2AX expression in human lungs 2 h post-LTx, which was reduced in murine grafts pretreated with the cathepsin B inhibitor CysC-Alb.

Overall, these results suggest the short-term use of the cystatin C–albumin fusion protein in the perfusion solution as a therapeutic opportunity to mitigate PGD immediately posttransplantation, where early intervention is critical. The observed protection of AT2 cells and reduction in acute injury suggest strong clinical potential in this setting. Although long-term use in BOS/CLAD remains a future direction, additional studies are needed to assess repeated dosing posttransplant, infection risk, and interactions with existing immunosuppressive therapies. However, that CysC-Alb not only facilitates longer graft preservation times but also potentially contributes to better graft function and health indicates its promise as a valuable tool in LTx procedures.

## Materials and Methods

### Human subjects

Lung transplant patients were included in the present study (Table S1), which was approved by the Institutional Review Board/Ethical Committees of the University Hospital of Ludwig Maximilian University Munich (protocol numbers #18-607 and #19-630). Informed consent was obtained from all patients, and experiments were conformed to the principles set out in the WMA Declaration of Helsinki and the Department of Health and Human Services Belmont Report. Bronchoalveolar lavage fluid (BALF) samples were obtained serially from the patients during bronchoscopy examination until the final day post-LTx and assigned a BOS score based upon lung function of the patient at the time of BALF.

### Production of recombinant CysC-Alb and Alb

**Figure S6.**
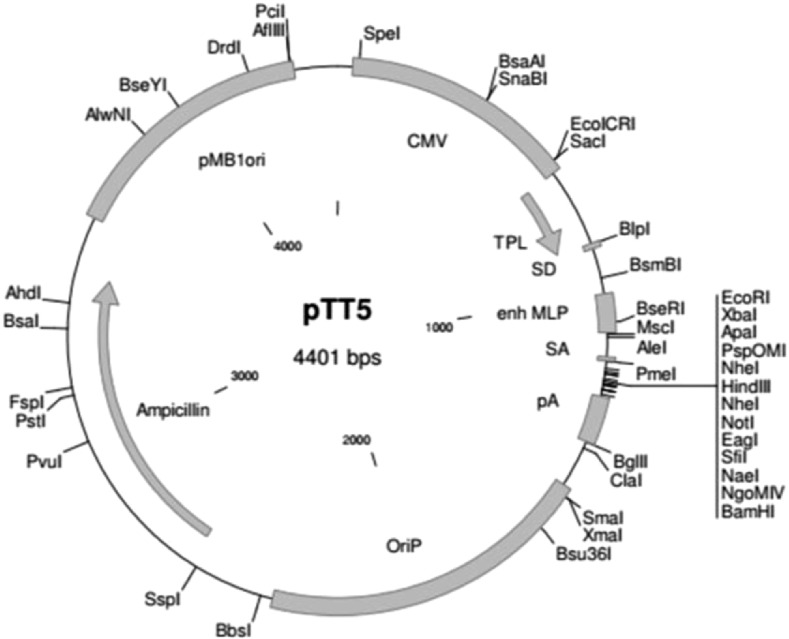
Vector map of the pTT5 plasmid used to express CysC fusion proteins in HEK293 cells. The vector contains an *E. coli* (pMB1ori)– and EBV (OriP)-specific origin of replication, a promoter of the cytomegalovirus (CMV), an adenovirus tripartite leader (TPL), a major late promoter (MLP), a rabbit beta-globin polyadenylation signal (pA), and an ampicillin resistance gene.

The cDNA of the CysC variant and Alb was subcloned in a modified version of the pTT5 expression vector (Fig S6), which already contained the reading frame of Igκ chain secretion signal peptide (METDTLLLWVLLLWVPGSTG) and a C-terminal his-tag of six histidines (obtained from Yves Durocher, NRC Biotechnology Research Institute). The his-tag remained intact and was not cleaved or degraded by proteases during expression in cell culture or purification. First, wild-type Alb was amplified from mouse liver lysates using the following primers: 5′-CGG​GGT​ACC​AGG​TTC​CAC​TGG​TGA​AGC​ACA​CAA​GAG​TGA-3′ and 5′-GAT​GAC​CGG​TGG​CTA​ACG​CGT​CTT​TGC​ATC​TAG​TGA​CA-3′. The PCR product was subcloned into the pTT5 vector using the KpnI restriction site at the 5′-end and the AgeI restriction site at the 3′-end located within the primers and the vector. Next, a synthetic cDNA (gBlock) encoding the modified mouse CysC sequence was ordered from Integrated DNA Technologies (IDT), USA. This double-stranded DNA was flanked by KpnI and StuI restriction sites and encodes the modified mature version of mouse CysC (Fig S8) starting at residue position 29 of the UniProt CysC sequence. The natural mouse CysC precursor includes a 20-residue signal peptide and an additional eight residues at the amino-terminus, which are not essential for interaction with cysteine proteases and were therefore omitted from the construct. The amino-terminal methionine at position 9 (numbering of the mature product, which is identical to residue 29 of the full-length polypeptide sequence) was replaced with a leucine at position 9, which is not removed by aminopeptidases during expression and secretion in HEK293 cells. In addition, a third disulfide bond (Cys47-Cys69) was introduced for monomeric stabilization (Kolodziejczyk et al, 2010). The CysC gBlock was flanked at the 5′-end by the KpnI site located at the 3′-end of the Igκ chain secretion signal peptide. The 3′-end of the CysC gBlock encoded a glycine linker and was followed by the 5′-sequence of mouse Alb up to the StuI site. The KpnI and StuI sites are unique within the pTT5-albumin plasmid allowing for directional insertion of the CysC fragment. The final cDNA construct was verified by sequence analysis (Supplemental Data 1).

Supplemental Data 1. Sequences of expressed proteins.

All recombinant proteins were expressed in HEK293-EBNA cells (obtained from Yves Durocher, NRC Biotechnology Research Institute) by transient transfection as described previously (L’Abbe et al, 2018). After expression, supernatants were clarified by centrifugation at 350*g* for 15 min and filtered through a 0.22-μm membrane (Millipore) to remove potential bacterial contamination. The clarified supernatants were then dialyzed overnight against 20 mM Na_2_HPO_4_ and 300 mM NaCl, pH 7.4, for CysC-Alb and against 20 mM sodium phosphate, pH 7, for Alb. Dialyzed samples were subsequently subjected to purification using HisTrap HP (CysC-Alb) and HiTrap Blue columns (Alb) according to the manufacturer’s instructions.

**Figure S7.**
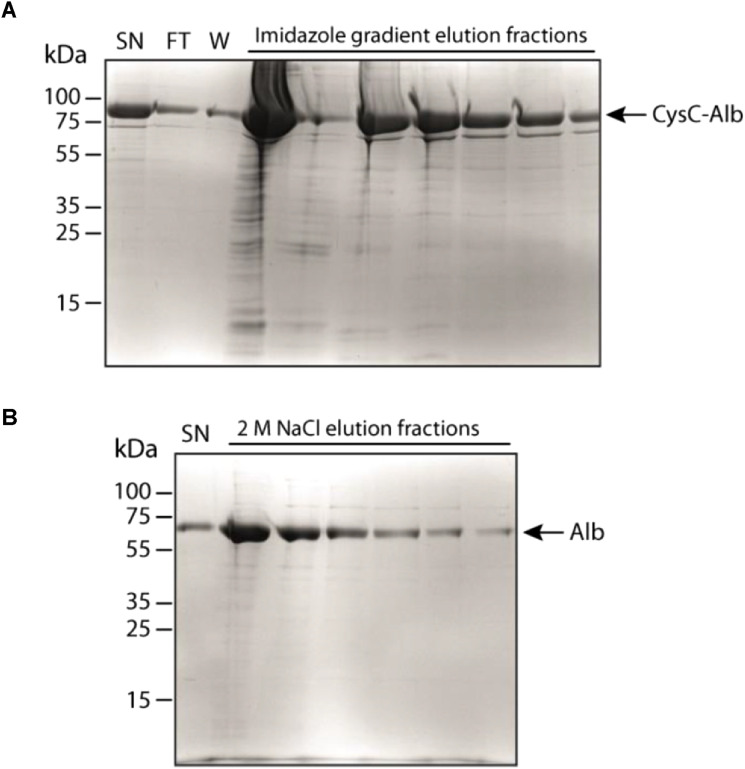
Production of CysC-Alb and Alb in HEK293E cells. Coomassie staining of cell culture supernatants and purified elution fractions after transfection of HEK293E cells with CysC-Alb or Alb. The CysC-Alb construct contains an Igκ signal peptide for secretion into the cell supernatant and a C-terminal his-tag for purification. The sequences are listed in Supplemental Data 1. **(A)** CysC-Alb was purified from 300 ml supernatant by Ni-NTA chromatography and eluted from the Ni-columns with an imidazole gradient (40, 60, 75, 150, 300, 500 mM, 1 M). **(B)** Alb was purified from 300 ml by HiTrap Blue HP affinity chromatography and eluted with 2 M NaCl. The volume of each elution fraction was 1.5 ml. 15 μl of the supernatant, the wash (with 20 mM Na_2_HPO_4_, 300 mM NaCl, pH 7.4), the flow-through, and the elution fractions were analyzed on a SDS–PAGE (SN, supernatant; F, flow-through; W, wash).

CysC-Alb and Alb were successfully expressed in HEK293E cells using the pTT5 expression vector. After 4 d, high levels of both proteins were detected in the culture supernatant. CysC-Alb was purified via Ni-NTA affinity chromatography using its C-terminal His-tag. The column was washed with binding buffer, and bound proteins were eluted with a linear imidazole gradient ranging from 40 mM to 1 M in binding buffer. Elution fractions were collected and analyzed by SDS–PAGE followed by Coomassie staining (Fig S7A). The elution profile of CysC-Alb showed minimal contamination with a faint band corresponding to albumin (67 kD), likely originating from dead cells. The fractions that contained the protein of interest were pooled and concentrated. Imidazole was removed by dialysis against the binding buffer. The highest yield of CysC-Alb obtained was 10 mg from 600 ml of cell culture. His-tagged Alb was purified via HiTrap Blue HP affinity chromatograph based on its interaction with the dye Cibacron Blue F3G-A (Fig S7B). The maximum yield achieved for Alb was 8 mg from 600 ml of cell culture medium.

Protein concentrations were determined spectrophotometrically by measuring absorbance at 280 nm by applying the Beer–Lambert law: c= (A280 × MW)/(є280 × l), where c = concentration; A = absorbance; MW = molecular weight; є = extinction coefficient; l = length of solution. Extinction coefficient of the recombinant proteins was calculated using the ProtParam tool (https://web.expasy.org/protparam/) by inserting the protein sequence obtained from the UniProt database (https://www.uniprot.org/).

**Figure S8.**
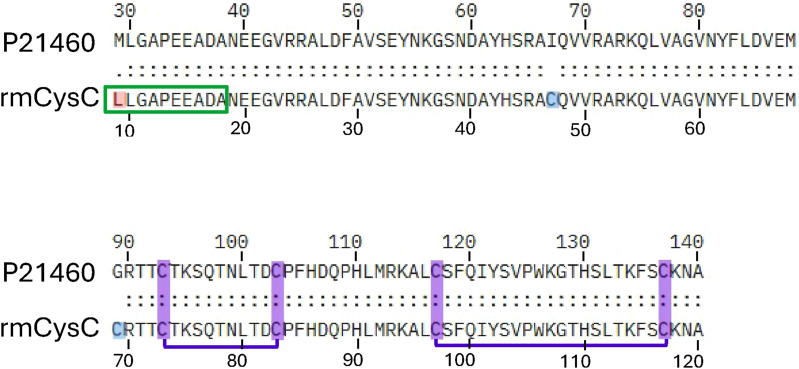
Sequence of recombinant mouse cystatin C (rmCysC) used in the CysC-Alb construct, in comparison with UniProt mouse CysC (P21460). Upper numbering starts with the first residue of the mRNA reading frame including the signal peptide of CysC from the UniProt sequence. The lower numbering starts from the ER-translocated (secreted) form of CysC. Sequence comparison starts at residue 29 of the UniProt sequence. The engineered N-terminal leucine residue for stability is highlighted in red. The two engineered cysteine residues to introduce a third disulfide bridge are highlighted in blue, connecting positions 47–69 of the secreted form. The two native disulfide bridges are highlighted in purple. The amino-terminal sequence of the recombinant protein was confirmed by Edman sequencing and indicated by a green box. Full sequence can be found in Supplemental Data 1.

N-terminal amino acids of CysC-Alb were determined by Edman sequencing (Fig S8), and functional activity was determined by determining the relative activity of 0.1 μM recombinant CatB in the presence of increasing concentrations of CysC-Alb (Fig S3A).

### Animal experiments

Female C57BL/6J mice were purchased from Charles River. The mice were housed with ad libitum access to food and water in the pathogen-free animal facility of the Helmholtz Zentrum München (Munich, Germany). All experiments were conducted under international guidelines and were approved by the local government and administration region for Upper Bavaria, Germany, under the project 55.2-1-54-2532.120.2015.

### Ex vivo lung perfusion (EVLP)

To perform EVLP in a small animal model, we used a modified version of an EVLP system previously described (Nelson et al, 2014). Three chambers were prepared: the inflow chamber (IC), the lung chamber (LC), and the outflow chamber (OC) (Fig S2). All three chambers were filled with Perfadex, and the IC was kept at 37°C in a water bath. The donor lung was ventilated via a 20G cannula (volume 70 μl/min, 80 strokes) and perfused through the pulmonary vein with a 26G cannula (0.35 ml/min). The ventilation cannula was connected to the bronchus, the inflow perfusion cannula to the pulmonary artery, and the outflow cannula to the pulmonary vein. After putting the lung in the LC, the perfusion system was started by turning on the peristaltic pump (0.35 ml/min) that is placed between the IC and the LC, and the ventilation system (80 strokes/min). Observed air bubbles in the pipeline were first removed using an air purge stopcock. The Perfadex solution containing CysC-Alb or Alb (40 μg/ml) was then perfused through the lung scaffold for 2 h. A second peristaltic pump between the LC and the OC was turned on to collect the perfusate in the OC. In total, 42 ml of perfusate was collected in the OC after 2-h EVLP.

### Orthotopic left LTx

Female C57BL/6 mice were purchased from The Jackson Laboratory. Orthotopic LTx procedures followed the protocol described by Krupnick et al with few modifications (Krupnick et al, 2009). No immunosuppression was applied to transplanted mice. C57BL/6 mice were used as donors and recipients of left lungs. Donors were anesthetized with an i.p. injection of ketamine/xylazine. The pulmonary artery, bronchus, and pulmonary vein were dissected from one another, before cuffing with 24-, 20-, and 22-gauge cuffs. The left lung graft was stored for 18 h in Perfadex at 4°C, with the addition of Alb or CysC-Alb (1 mg/ml); afterward the graft was perfused for 2 h via EVLP. EVLP was performed via three chambers: the inflow chamber (IC), the lung chamber (LC), and the outflow chamber (OC). All three chambers were filled with Perfadex, and the IC was gradually warmed up to 37°C. The donor lung was connected via a tracheal cannula to the ventilator (80 strokes/min) and perfused through the pulmonary vein with a 26G cannula, connected to the IC. The perfusion system is started by turning on the peristaltic pump (0.35 ml/min). The perfusion flow was kept free from air bubbles. The perfusion solution containing either CysC-Alb or Alb (40 μg/ml) was perfused through the lung scaffold for 2 h. A second peristaltic pump was used to collect the perfusate in the OC overtime. A total of 42 ml of perfusate was collected after 2-h EVLP. The left lung was implanted for 4 h in a recipient mouse, to simulate ischemia–reperfusion damage. After 4 h, the recipient was anesthetized with a mixture of medetomidine/midazolam/fentanyl (1/0.05/0.02 mg/kg), then intubated (300 μl room air/120 strokes/min); an abdominal incision was executed; and the chest was opened to expose the cardiopulmonary system. The right bronchus and pulmonary vein were clamped for 3 min, and the ventilation setting was modified (75 μl room air/120 strokes/min) to determine the oxygenation function of the transplanted left lung by measuring the pO2 [%] of the blood from the left ventricle (ABL80 FLEX CO-OX Analyzer, Radiometer). The upper part of the lung was fixed with 4% PFA for histology analysis, and the lower part was used for protein analysis.

### ELISA cystatin C immunoassay

The concentration of human cystatin C was measured using the Human Cystatin C (CST3) Sandwich ELISA Kit from Invitrogen Thermo Fisher Scientific (Cat. No. EHCST3). Microtiter plates precoated with an anti-human cystatin C antibody were used. A 100 μl aliquot of the BALF sample was added to each well and incubated overnight at 4°C in a gently shaking incubator. After incubation, the plates were washed four times with 1X Assay Buffer (provided in the kit).

Next, the biotin-conjugated anti-human cystatin C detection antibody was added and incubated for 1 h at RT, followed by incubation with streptavidin–HRP conjugate solution for 45 min. After additional washing steps, TMB substrate (3,3′,5,5′-tetramethylbenzidine) was added and incubated for 30 min in the dark. The reaction was stopped using the stop solution provided, and absorbance was measured at 450 nm using a microplate reader (Infinite 200 PRO; TECAN).

### Immunohistochemical analysis (DAB)

Tissue sections were deparaffinized in two cycles of xylene and rehydrated through a descending ethanol series. Antigen retrieval was performed by heating the slides in an automated pressure cooker using 10 mM EDTA buffer (pH 6.5). Endogenous peroxidase activity was quenched by incubating the sections in 3% hydrogen peroxide (prepared from 30% stock, H1009; Sigma-Aldrich) in methanol. Slides were then washed and rehydrated with PBS containing 0.1% Tween-20 (PBST) for 10 min. To block endogenous biotin, slides were treated with avidin solution (Avidin/Biotin Blocking Kit, SP-2001; Vector Laboratories) for 15 min, followed by a PBST wash and incubation with biotin solution from the same kit for another 15 min, then washed again. Nonspecific binding was blocked using 3% BSA in PBS (PBSA) for 1 h. Sections were then incubated overnight at 4°C with the following primary antibodies: cystatin C rabbit monoclonal antibody (ab109508; Abcam), γ-H2AX rabbit monoclonal antibody (#9718; Cell Signaling Technology), and ACSL4 (F6T3Z) rabbit monoclonal antibody (#38493; Cell Signaling Technology). After washing with PBST, slides were incubated with a HRP-conjugated anti-rabbit IgG from Vector Laboratories for 30 min at RT. Detection was performed using a DAB substrate kit (ImmPACT DAB, SK-4105; Vector Laboratories), and counterstaining was done with hematoxylin. Finally, the slides were dehydrated, mounted, and scanned using a slide scanner (Axio Imager M2; Zeiss).

### Western blot

The total protein concentration of whole lung lysate prepared in RIPA lysis buffer (50 mM Tris, 150 mM NaCl, 1 mM EDTA, 0.5% [wt/vol] deoxycholic acid, 0.1% [wt/vol] SDS, 0.5% [vol/vol] Nonidet P-40, pH 8.0) was determined by Pierce BCA Protein Assay Kit. 30 μg protein was separated on 12% SDS–PAGE under denaturing conditions and transferred to a polyvinylidene difluoride (PVDF) membrane. Free sites on the membrane were blocked by incubation with 5% milk powder (Bio-Rad). Membranes were then incubated with a rabbit primary anti-cystatin C monoclonal antibody (1:500, #ab109508; Abcam) at 4 °C overnight, and normalized to β-actin levels (anti-β-actin-peroxidase–conjugated mouse monoclonal antibody, AC-15, Cat. No. A3854, 1:50,000, Sigma-Aldrich). Detection of bound primary antibodies was performed by ECL after α-mouse (1:3,000, NA931; GE HealthCare) secondary antibody incubation.

### Statistical analysis

The data were presented as the mean ± SD, including sample size and number of repeats, as stated in the figure legends. All studies involving more than two groups were compared using either a one-way ANOVA followed by a Bonferroni posttest or a Kruskal–Wallis test with Dunn’s multiple comparisons test if the data were not normally distributed. *t* tests were used to compare the findings between two groups. *P*-values of less than 0.05 were considered significant. Analyses were performed using GraphPad Prism 9 software.

## Supplementary Material

Reviewer comments

## Data Availability

Source data are provided within this article as supplementary Source Data Files. Sequencing of the constructs is provided in Supplemental Data 1. All data are available upon request to the corresponding author (oender.yildirim@helmholtz-munich.de).
