# Supplementary material for: Cystatin C prevents tissue injury after lung transplantation
Source: Life Sci Alliance. 2025 Dec 16;9(2):e202503312. doi: 10.26508/lsa.202503312 (PMC12709050; doi:10.26508/lsa.202503312)
Supplement: Supplementary file 1 [file LSA-2025-03312_TableS1.docx]

|  | **Stable** | **BOS** |
| --- | --- | --- |
| **Number of patients** | 24 | 39 |
| **Age at LTx (years)** | 45·27 ± 14·02 | 46·31 ± 13·46 |
| **Sex (F/M)** | 13 F / 11 M | 11 F / 14 M |
| **Underlying disease** | Bronchiolitis = 2  Bronchiectasis = 1  COPD = 4  CF = 3  ILD = 13  PH = 1 | Bronchiolitis = 2  Bronchiectasis = 1  COPD = 11  CF = 4  ILD = 19  PH = 2 |
| **Procedure** | All double LTx | All double LTx |
| **Days after LTx (of final BALF)** | 252 ± 240 | 1765 ± 985 |
| **Grade at BALF** | No BOS | Pre BOS n = 28  BOS 1 n = 35  BOS 2 n = 33  BOS 3 n = 31 |

**Table S1: Patient characteristics of lung transplant recipients.**

Patients were clustered according to their gender (F) for female and (M) for male, as well as the underlying diseases; COPD referred to chronic obstructive pulmonary disease, ILD referred to interstitial lung diseases, CF referred to cystic fibrosis, and PH referred to pulmonary hypertension. BALF was serially obtained from patients up until final day post LTx and assigned the BOS score based upon lung function of the patient at the time of BALF.
