# Supplementary material for: Cystatin C prevents tissue injury after lung transplantation
Source: Life Sci Alliance. 2025 Dec 16;9(2):e202503312. doi: 10.26508/lsa.202503312 (PMC12709050; doi:10.26508/lsa.202503312)
Supplement: Supplementary file 8 [file LSA-2025-03312_Supplemental_Data_1.pdf]

Sequences of expressed proteins

The amino acid sequence is represented by a single letter code. The sequence begins with the Igk signal peptide highlighted in a grey box. CysC is marked by a red box and the glycine linker purple. The Alb sequence is unboxed. The C-terminal His-tag is marked by a blue box and the stop codon is represented by a star (*). Restriction sites utilized for sub-cloning are indicated.

pTT5-CysC-Alb

```
                                                                      KpnI
   1 atg gag aca gac aca ctc ctg cta tgg gta ctg ctg ctc tgg gta cct ggc tca acc gga
   1 M   E   T   D   T   L   L   L   W   V   L   L   L   W   V   P   G   S   T   G
  61 ctg ctg ggg gct ccc gag gaa gcg gat gct aac gag gaa ggc gtc aga agg gcc ttg gat
  21 L   L   G   A   P   E   E   A   D   A   N   E   E   G   V   R   R   A   L   D
 121 ttc gct gtc tca gag tat aat aag ggt tcc aac gac gcg tat cat agc agg gca tgt cag
  41 F   A   V   S   E   Y   N   K   G   S   N   D   A   Y   H   S   R   A   C   Q
 181 gtt gta cgc gca cgg aaa caa ctc gtc gca ggc gtt aac tac ttc ttg gat gtg gaa atg
  61 V   V   R   A   R   K   Q   L   V   A   G   V   N   Y   F   L   D   V   E   M
 241 tgc cgg acc acg tgc acc aag agt cag acc aat ctt acc gac tgc cca ttt cat gac caa
  81 C   R   T   T   C   T   K   S   Q   T   N   L   T   D   C   P   F   H   D   Q
 301 ccc cac ctt atg aga aag gcc ttg tgc agt ttc cag ata tat agc gta ccc tgg aag gga
 101 P   H   L   M   R   K   A   L   C   S   F   Q   I   Y   S   V   P   W   K   G
 361 act cat agc ctg acg aag ttt agc tgt aaa aac gct ggt gga ggc ggc acc ggt gaa gca
 121 T   H   S   L   T   K   F   S   C   K   N   A   G   G   G   G   T   G   E   A
                                                                               StuI
 421 cac aag agt gag atc gcc cat cgg tat aat gat ttg gga gaa caa cat ttc aaa ggc cta
 141 H   K   S   E   I   A   H   R   Y   N   D   L   G   E   Q   H   F   K   G   L
 481 gtc ctg att gcc ttt tcc cag tat ctc cag aaa agc tca tac gat gag cat gcc aaa tta
 161 V   L   I   A   F   S   Q   Y   L   Q   K   S   S   Y   D   E   H   A   K   L
 541 gtg cag gaa gta aca gac ttt gca aag acg tgt gtt gcc gat gag tct gcc gcc aac tgt
 181 V   Q   E   V   T   D   F   A   K   T   C   V   A   D   E   S   A   A   N   C
 601 gac aaa tcc ctt cac act ctt ttt gga gat aag ttg tgt gcc att cca aac ctc cgt gaa
 201 D   K   S   L   H   T   L   F   G   D   K   L   C   A   I   P   N   L   R   E
 661 aac tat ggt gaa ctg gct gac tgc tgt aca aaa caa gag ccc gaa aga aac gaa tgt ttc
 221 N   Y   G   E   L   A   D   C   C   T   K   Q   E   P   E   R   N   E   C   F
 721 ctg caa cac aaa gat gac aac ccc agc ctg cca cca ttt gaa agg cca gag gct gag gcc
 241 L   Q   H   K   D   D   N   P   S   L   P   P   F   E   R   P   E   A   E   A
 781 atg tgc acc tcc ttt aag gaa aac cca acc acc ttt atg gga cac tat ttg cat gaa gtt
 261 M   C   T   S   F   K   E   N   P   T   T   F   M   G   H   Y   L   H   E   V
 841 gcc aga aga cat cct tat ttc tat gcc cca gaa ctt ctt tac tat gct gag cag tac aat
 281 A   R   R   H   P   Y   F   Y   A   P   E   L   L   Y   Y   A   E   Q   Y   N
 901 gag att ctg acc cag tgt tgt gca gag gct gac aag gaa agc tgc ctg acc ccg aag ctt
 301 E   I   L   T   Q   C   C   A   E   A   D   K   E   S   C   L   T   P   K   L
 961 gat ggt gtg aag gag aaa gca ttg gtc tca tcg gtc cgt cag aga atg aag tgc tcc agt
 321 D   G   V   K   E   K   A   L   V   S   S   V   R   Q   R   M   K   C   S   S
1021 atg cag aag ttt gga gag aga gct ttt aaa gca tgg gca gta gct cgt ctg agc cag aca
 341 M   Q   K   F   G   E   R   A   F   K   A   W   A   V   A   R   L   S   Q   T
1081 ttc ccc aat gct gac ttt gca gaa atc acc aaa ttg gca aca gac ctg acc aaa gtc aac
 361 F   P   N   A   D   F   A   E   I   T   K   L   A   T   D   L   T   K   V   N
```

pTT5-CysC-Alb continued

```
1141 aag gag tgc tgc cat ggt gac ctg ctg gaa tgc gca gat gac agg gcg gaa ctt gcc aag
 381 K   E   C   C   H   G   D   L   L   E   C   A   D   D   R   A   E   L   A   K
1201 tac atg tgt gaa aac cag gcg act atc tcc agc aaa ctg cag act tgc tgc gat aaa cca
 401 Y   M   C   E   N   Q   A   T   I   S   S   K   L   Q   T   C   C   D   K   P
1261 ctg ttg aag aaa gcc cac tgt ctt agt gag gtg gag cat gac acc atg cct gct gat ctg
 421 L   L   K   K   A   H   C   L   S   E   V   E   H   D   T   M   P   A   D   L
1321 cct gcc att gct gct gat ttt gtt gag gac cag gaa gtg tgc aag aac tat gct gag gcc
 441 P   A   I   A   A   D   F   V   E   D   Q   E   V   C   K   N   Y   A   E   A
1381 aag gat gtc ttc ctg ggc acg ttc ttg tat gaa tat tca aga aga cac cct gat tac tct
 461 K   D   V   F   L   G   T   F   L   Y   E   Y   S   R   R   H   P   D   Y   S
1441 gta tcc ctg ttg ctg aga ctt gct aag aaa tat gaa gcc act ctg gaa aag tgc tgc gct
 481 V   S   L   L   L   R   L   A   K   K   Y   E   A   T   L   E   K   C   C   A
1501 gaa gcc aat cct ccc gca tgc tac ggc aca gtg ctt gct gaa ttt cag cct ctt gta gaa
 501 E   A   N   P   P   A   C   Y   G   T   V   L   A   E   F   Q   P   L   V   E
1561 gag cct aag aac ttg gtc aaa acc aac tgt gat ctt tac gag aag ctt gga gaa tat gga
 521 E   P   K   N   L   V   K   T   N   C   D   L   Y   E   K   L   G   E   Y   G
1621 ttc caa aat gcc att cta gtt cgc tac acc cag aaa gca cct cag gtg tca acc cca act
 541 F   Q   N   A   I   L   V   R   Y   T   Q   K   A   P   Q   V   S   T   P   T
1681 ctc gtg gag gct gca aga aac cta gga aga gtg ggc acc aag tgt tgt aca ctt cct gaa
 561 L   V   E   A   A   R   N   L   G   R   V   G   T   K   C   C   T   L   P   E
1741 gat cag aga ctg cct tgt gtg gaa gac tat ctg tct gca atc ctg aac cgt gtg tgt ctg
 581 D   Q   R   L   P   C   V   E   D   Y   L   S   A   I   L   N   R   V   C   L
1801 ctg cat gag aag acc cca gtg agt gag cat gtt acc aag tgc tgt agt gga tcc ctg gtg
 601 L   H   E   K   T   P   V   S   E   H   V   T   K   C   C   S   G   S   L   V
1861 gaa agg cgg cca tgc ttc tct gct ctg aca gtt gat gaa aca tac gta ccc aaa gag ttt
 621 E   R   R   P   C   F   S   A   L   T   V   D   E   T   Y   V   P   K   E   F
1921 aaa gct gag acc ttc acc ttc cac tct gat atc tgc aca ctt cca gag aag gag aag cag
 641 K   A   E   T   F   T   F   H   S   D   I   C   T   L   P   E   K   E   K   Q
1981 att aag aaa caa acg gct ctt gct gag ctg gtg aag cac aag ccc aag gct aca gcg gag
 661 I   K   K   Q   T   A   L   A   E   L   V   K   H   K   P   K   A   T   A   E
2041 caa ctg aag act gtc atg gat gac ttt gca cag ttc ctg gat aca tgt tgc aag gct gct
 681 Q   L   K   T   V   M   D   D   F   A   Q   F   L   D   T   C   C   K   A   A
2101 gac aag gac acc tgc ttc tcg act gag ggt cca aac ctt gtc act aga tgc aaa gac gcg
 701 D   K   D   T   C   F   S   T   E   G   P   N   L   V   T   R   C   K   D   A
2161 tta gcc acc ggt cat cat cac cat cac cat tga
 721 L   A   T   G   H   H   H   H   H   H   *
```

AgeI

pTT5-Alb

```
                                                                  KpnI
1 atg gag aca gac aca ctc ctg cta tgg gta ctg ctg ctc tgg gta cca ggt tcc act ggt
1 M   E   T   D   T   L   L   L   W   V   L   L   L   W   V   P   G   S   T   G
61 gaa gca cac aag agt gag atc gcc cat cgg tat aat gat ttg gga gaa caa cat ttc aaa
StuI 21 E   A   H   K   S   E   I   A   H   R   Y   N   D   L   G   E   Q   H   F   K
121 ggc cta gtc ctg att gcc ttt tcc cag tat ctc cag aaa tgc tca tac gat gag cat gcc
41 G   L   V   L   I   A   F   S   Q   Y   L   Q   K   C   S   Y   D   E   H   A
181 aaa tta gtg cag gaa gta aca gac ttt gca aag acg tgt gtt gcc gat gag tct gcc gcc
61 K   L   V   Q   E   V   T   D   F   A   K   T   C   V   A   D   E   S   A   A
241 aac tgt gac aaa tcc ctt cac act ctt ttt gga gat aag ttg tgt gcc att cca aac ctc
81 N   C   D   K   S   L   H   T   L   F   G   D   K   L   C   A   I   P   N   L
301 cgt gaa aac tat ggt gaa ctg gct gac tgc tgt aca aaa caa gag ccc gaa aga aac gaa
101 R   E   N   Y   G   E   L   A   D   C   C   T   K   Q   E   P   E   R   N   E
361 tgt ttc ctg caa cac aaa gat gac aac ccc agc ctg cca cca ttt gaa agg cca gag gct
121 C   F   L   Q   H   K   D   D   N   P   S   L   P   P   F   E   R   P   E   A
421 gag gcc atg tgc acc tcc ttt aag gaa aac cca acc acc ttt atg gga cac tat ttg cat
141 E   A   M   C   T   S   F   K   E   N   P   T   T   F   M   G   H   Y   L   H
481 gaa gtt gcc aga aga cat cct tat ttc tat gcc cca gaa ctt ctt tac tat gct gag cag
161 E   V   A   R   R   H   P   Y   F   Y   A   P   E   L   L   Y   Y   A   E   Q
541 tac aat gag att ctg acc cag tgt tgt gca gag gct gac aag gaa agc tgc ctg acc ccg
181 Y   N   E   I   L   T   Q   C   C   A   E   A   D   K   E   S   C   L   T   P
601 aag ctt gat ggt gtg aag gag aaa gca ttg gtc tca tct gtc cgt cag aga atg aag tgc
201 K   L   D   G   V   K   E   K   A   L   V   S   S   V   R   Q   R   M   K   C
661 tcc agt atg cag aag ttt gga gag aga gct ttt aaa gca tgg gca gta gct cgt ctg agc
221 S   S   M   Q   K   F   G   E   R   A   F   K   A   W   A   V   A   R   L   S
721 cag aca ttc ccc aat gct gac ttt gca gaa atc acc aaa ttg gca aca gac ctg acc aaa
241 Q   T   F   P   N   A   D   F   A   E   I   T   K   L   A   T   D   L   T   K
781 gtc aac aag gag tgc tgc cat ggt gac ctg ctg gaa tgc gca gat gac agg gcg gaa ctt
261 V   N   K   E   C   C   H   G   D   L   L   E   C   A   D   D   R   A   E   L
841 gcc aag tac atg tgt gaa aac cag gcg act atc tcc agc aaa ctg cag act tgc tgc gat
281 A   K   Y   M   C   E   N   Q   A   T   I   S   S   K   L   Q   T   C   C   D
901 aaa cca ctg ttg aag aaa gcc cac tgt ctt agt gag gtg gag cat gac acc atg cct gct
301 K   P   L   L   K   K   A   H   C   L   S   E   V   E   H   D   T   M   P   A
961 gat ctg cct gcc att gct gct gat ttt gtt gag gac cag gaa gtg tgc aag aac tat gct
321 D   L   P   A   I   A   A   D   F   V   E   D   Q   E   V   C   K   N   Y   A
1021 gag gcc aag gat gtc ttc ctg ggc acg ttc ttg tat gaa tat tca aga aga cac cct gat
341 E   A   K   D   V   F   L   G   T   F   L   Y   E   Y   S   R   R   H   P   D
1081 tac tct gta tcc ctg ttg ctg aga ctt gct aag aaa tat gaa gcc act ctg gaa aag tgc
361 Y   S   V   S   L   L   L   R   L   A   K   K   Y   E   A   T   L   E   K   C
1141 tgc gct gaa gcc aat cct ccc gca tgc tac ggc aca gtg ctt gct gaa ttt cag cct ctt
381 C   A   E   A   N   P   P   A   C   Y   G   T   V   L   A   E   F   Q   P   L
```

pTT5-Alb continued

```
1201 gta gaa gag cct aag aac ttg gtc aaa acc aac tgt gat ctt tac gag aag ctt gga gaa
 401 V   E   E   P   K   N   L   V   K   T   N   C   D   L   Y   E   K   L   G   E
1261 tat gga ttc caa aat gcc att cta gtt cgc tac acc cag aaa gca cct cag gtg tca acc
 421 Y   G   F   Q   N   A   I   L   V   R   Y   T   Q   K   A   P   Q   V   S   T
1321 cca act ctc gtg gag gct gca aga aac cta gga aga gtg ggc acc aag tgt tgt aca ctt
 441 P   T   L   V   E   A   A   R   N   L   G   R   V   G   T   K   C   C   T   L
1381 cct gaa gat cag aga ctg cct tgt gtg gaa gac tat ctg tct gca atc ctg aac cgt gtg
 461 P   E   D   Q   R   L   P   C   V   E   D   Y   L   S   A   I   L   N   R   V
1441 tgt ctg ctg cat gag aag acc cca gtg agt gag cat gtt acc aag tgc tgt agt gga tcc
 481 C   L   L   H   E   K   T   P   V   S   E   H   V   T   K   C   C   S   G   S
1501 ctg gtg gaa agg cgg cca tgc ttc tct gct ctg aca gtt gat gaa aca tat gtc ccc aaa
 501 L   V   E   R   R   P   C   F   S   A   L   T   V   D   E   T   Y   V   P   K
1561 gag ttt aaa gct gag acc ttc acc ttc cac tct gat atc tgc aca ctt cca gag aag gag
 521 E   F   K   A   E   T   F   T   F   H   S   D   I   C   T   L   P   E   K   E
1621 aag cag att aag aaa caa acg gct ctt gct gag ctg gtg aag cac aag ccc aag gct aca
 541 K   Q   I   K   K   Q   T   A   L   A   E   L   V   K   H   K   P   K   A   T
1681 gcg gag caa ctg aag act gtc atg gat gac ttt gca cag ttc ctg gat aca tgt tgc aag
 561 A   E   Q   L   K   T   V   M   D   D   F   A   Q   F   L   D   T   C   C   K
1741 gct gct gac aag gac acc tgc ttc tcg act gag ggt cca aac ctt gtc act aga tgc aaa
 581 A   A   D   K   D   T   C   F   S   T   E   G   P   N   L   V   T   R   C   K
1801 gac gcg tta gcc a|cc ggt cat cat cac cat cac cat tga
 601 D   A   L   A   T   G   H   H   H   H   H   H   *
                    |
                  AgeI
```
